# Renal Insufficiency and Early Bystander CPR Predict In-Hospital Outcomes in Cardiac Arrest Patients Undergoing Mild Therapeutic Hypothermia and Cardiac Catheterization: Return of Spontaneous Circulation, Cooling, and Catheterization Registry (ROSCCC Registry)

**DOI:** 10.1155/2016/8798261

**Published:** 2016-01-18

**Authors:** Anjala Chelvanathan, David Allen, Hilary Bews, John Ducas, Kunal Minhas, Minh Vo, Malek Kass, Amir Ravandi, James W. Tam, Davinder S. Jassal, Farrukh Hussain

**Affiliations:** ^1^Section of Cardiology, Department of Internal Medicine, University of Manitoba, Winnipeg, MB, Canada R2H 2A6; ^2^Institute of Cardiovascular Sciences, St. Boniface Research Centre, University of Manitoba, Winnipeg, MB, Canada R2H 2A6; ^3^Section of Oncology, Department of Internal Medicine, University of Manitoba, Winnipeg, MB, Canada R2H 2A6; ^4^Department of Radiology, University of Manitoba, Winnipeg, MB, Canada R2H 2A6

## Abstract

*Objective.* Out of hospital cardiac arrest (OHCA) patients are a critically ill patient population with high mortality. Combining mild therapeutic hypothermia (MTH) with early coronary intervention may improve outcomes in this population. The aim of this study was to evaluate predictors of mortality in OHCA patients undergoing MTH with and without cardiac catheterization.* Design.* A retrospective cohort of OHCA patients who underwent MTH with catheterization (MTH + C) and without catheterization (MTH + NC) between 2006 and 2011 was analyzed at a single tertiary care centre. Predictors of in-hospital mortality and neurologic outcome were determined.* Results.* The study population included 176 patients who underwent MTH for OHCA. A total of 66 patients underwent cardiac catheterization (MTH + C) and 110 patients did not undergo cardiac catheterization (MTH + NC). Immediate bystander CPR occurred in approximately half of the total population. In the MTH + C and MTH + NC groups, the in-hospital mortality was 48% and 78%, respectively. The only independent predictor of in-hospital mortality for patients with MTH + C, after multivariate analysis, was baseline renal insufficiency (OR = 8.2, 95% CI 1.8–47.1, and* p* = 0.009).* Conclusion.* Despite early cardiac catheterization, renal insufficiency and the absence of immediate CPR are potent predictors of death and poor neurologic outcome in patients with OHCA.

## 1. Introduction

Over 350,000 out of hospital cardiac arrests (OHCA) occur each year in the United States, with around 45,000 cardiac arrests occurring each year in Canada [1]. Despite nearly 40 years of promotion of prehospital Advanced Cardiac Life Support (ACLS), long-term survival rates following OHCA remain dismal [2, 3]. In a recent meta-analysis, the aggregate survival rate for OHCA across various populations was reported at less than 8% [3]. In addition, postcardiac arrest complications, including severe anoxic brain injury (ABI), contributes to high morbidity and mortality rates for patients initially undergoing successful resuscitation [1]. As such, it remains an important goal to develop therapeutic strategies to improve survival in this patient population.

Mild therapeutic hypothermia (MTH) and targeted temperature management (TTM) afford long-term survival and neurologic benefits to comatose survivors of arrhythmia-induced cardiac arrest [1, 3–6]. More recently, studies have investigated the combination of early interventional strategies with MTH as a means of further improving long-term survival in resuscitated cardiac arrest patients with evolving evidence of ST elevation myocardial infarction (STEMI). A review of four nonrandomized case series involving STEMI patients who were successfully resuscitated from cardiac arrest and treated with both MTH and early coronary intervention demonstrated overall favorable outcomes [7–10]. Numerous other case series have illustrated that early coronary angiography and percutaneous coronary intervention (PCI), combined with MTH, produce the highest long-term survival rates among patients who remain comatose after resuscitated cardiac arrest [11–14]. As a result, in the ACCF/AHA 2013 STEMI Guidelines, MTH was endorsed as a class 1, level of evidence B recommendation [15]. Furthermore, immediate angiography and PCI as indicated were recommended in resuscitated OHCA patients whose initial ECG shows ST-segment elevation [15].

The objective of this retrospective study was to investigate the outcomes and predictors of mortality in individuals resuscitated from cardiac arrest who underwent MTH both with and without cardiac catheterization.

## 2. Methods

### 2.1. Study Design

A single-centre retrospective cohort study examining all consecutive adult patients who were successfully resuscitated following cardiac arrest who underwent either MTH with catheterization (MTH + C) or MTH without cardiac catheterization (MTH + NC) between January 1, 2006, and September 30, 2011, was performed. MTH was used at the discretion of the attending physician and the decision regarding need for cardiac catheterization was made by the treating cardiologist or intensivist. MTH was induced and maintained through a combination of surface cooling blankets and ice packs. Local institutional protocol stipulates that MTH should be initiated as soon as possible after return of spontaneous circulation (ROSC) to achieve a core temperature of 32–34°C within 6–8 hrs. This core temperature should be maintained for 24 hrs and followed by subsequent passive rewarming. The study was approved by the University of Manitoba and St. Boniface General Hospital research ethics boards.

### 2.2. Data Collection

Subjects were identified by searching the ICU quality-assurance database for an International Classification of Diseases-9 discharge diagnosis of cardiac arrest in addition to cointerrogation of the MACLAB cardiac catheterization database. Detailed demographics and prehospital data pertaining to the cardiac arrest, including location of cardiac arrest, duration of down time, bystander CPR, time to ROSC, and initial arrest rhythm, were collected. As cardiac rhythm monitoring was not always available during the pulseless period, the initial arrest rhythm was the first rhythm recorded either during or after the cardiac arrest. Cooling protocol and time data were noted. Detailed data on cardiac interventions were recorded, including coronary anatomy, stent placement, coronary artery bypass grafting (CABG), mechanical circulatory support (MCS), and intra-aortic balloon pump (IABP) placement. Detailed data on complications within 1 week of hospitalization, in addition to patient outcomes, including in-hospital mortality, were abstracted.

### 2.3. Definitions

Cardiac arrest was defined as the absence of signs of circulation with the concomitant appearance of unconsciousness, apnea, or gasping and receipt of chest compressions or defibrillation for a pulseless arrhythmia as determined by a health care worker. Multivessel coronary disease was defined as ≥2 main (left anterior descending, right coronary artery, or circumflex) vessels with ≥70% stenosis. Early coronary angiogram was defined as receiving a coronary angiogram within 12 hours of arrival. A successful PCI was defined as residual stenosis ≤ 20% with TIMI 3 flow. Slow flow was defined as TIMI ≤ 2 flow in the intervened vessel. Angiographic, TIMI flow, and procedural success analysis was performed by local angiographic operators according to the aforementioned set criteria; there was no core lab available for analysis. The decision for PCI or CABG was at the operator's discretion, given the nature of a retrospective analysis.

Left ventricular ejection fraction (LVEF) was quantified using standard two-dimensional transthoracic echocardiography (TTE) or left ventriculography, whichever was available closer to index catheterization. The timing for TTE was variable, as this was a retrospective analysis and could include pre- or postrevascularization studies.

Cardiogenic shock was defined as systolic blood pressure (SBP) ≤ 90 mmHg for >30 minutes or the requirement of vasopressor/inotropic support to maintain SBP > 90 mmHg in addition to evidence of end organ hypoperfusion. Baseline renal insufficiency was defined as creatinine clearance (CrCl) < 60 mL/min. CrCl was calculated using the standard Cockgroft-Gault equation (CrCl = (140 − age) × wt (kg) ×  *F*/(plasma creatinine × 0.8136), where *F* = 1 if male and 0.85 if female). Report of anoxic brain injury (ABI) required chart documentation by the intensivist or neurologist in the ICU and/or CT brain evidence of ABI with a clinical agreement note.

The Glasgow-Pittsburgh Cerebral Performance Category (CPC) score was utilized to assess neurologic recovery [16]. The best CPC score achieved at hospital discharge was recorded by the treating neurologist or allied health personnel. A CPC score of 1 or 2 represented favorable functional neurologic recovery and was therefore defined as good neurologic outcome. A CPC score of 3, 4, or 5 reflected poor neurologic recovery.

### 2.4. Statistics

Descriptive statistical methods were used to summarize data. A negative binomial logistic regression model was utilized to identify univariate and multivariate predictors of in-hospital mortality. All univariate predictors with a *p* value < 0.05 were considered significant and were included into a stringent multivariable model to prevent model instability given our small sample size. SAS version 9.1.2 software was utilized to perform all analyses. Prespecified subgroup analyses were performed to identify univariate and multivariate predictors of good neurologic outcome.

## 3. Results

### 3.1. Study Population

A total of 176 consecutive patients (mean age 61 ± 13 years, 129 males) with a documented cardiac arrest who were admitted to a single tertiary care ICU and underwent MTH were the initial study population. A total of 66 patients underwent MTH + C at the discretion of the treating cardiologist or intensivist; the remaining 110 patients did not undergo cardiac catheterization (MTH + NC). Baseline demographics of the two study groups are outlined in Table 1. Despite the majority of patients in the MTH + C group having experienced a witnessed cardiac arrest (92%), only half of patients (55%) received immediate bystander CPR. Similarly, in the MTH + NC group, approximately 75% experienced a cardiac arrest, of which 45% received immediate bystander CPR. The mean duration of bystander CPR was similar at 6.4 ± 4.1 minutes and 7.5 ± 4.0 minutes for the MTH + C and MTH + NC groups, respectively. The median interval from the occurrence of cardiac arrest to ROSC was also similar at 28 ± 15 minutes and 24 ± 15 minutes for the MTH + C and MTH + NC groups, respectively. In the majority of patients who underwent MTH + C, ventricular fibrillation (VF)/pulseless ventricular tachycardia (VT) (92%) was the initial resuscitated cardiac rhythm, with 68% demonstrating evidence of STEMI. In patients who underwent MTH + NC, the initial cardiac rhythm was PEA or asystole in 65% of cases. The majority of patients in the entire population (>80%) were in cardiogenic shock requiring vasopressor or inotropic support within the first week of resuscitation following cardiac arrest. Baseline renal insufficiency was present in 26% of patients in the MTH + C group as compared to 11% in patients in the MTH + NC group (*p* = 0.8).

### 3.2. Mild Therapeutic Hypothermia (MTH)

The parameters for MTH are listed in Table 1. The mean time from ROSC to initiation of MTH was 277 ± 110 minutes (4.6 ± 1.8 hrs) for the catheterization group and 211 ± 146 minutes (3.5 ± 2.4 hrs) for the no catheterization group. An average time of 252 ± 174 minutes (4.2 ± 2.9 hrs) and 312 ± 465 minutes (5.2 ± 7.8 hrs) for the MTH + C and MTH + NC groups, respectively, from cooling initiation was required to achieve the target cooling temperature between 32 and 34°C. Total time at target cooling temperature was 1671 ± 410 minutes (27.9 ± 6.8 hrs) for the MTH + C group and 1625 ± 551 minutes (27.1 ± 9.2 hrs) for the MTH + NC group. MTH was initiated prior to cardiac catheterization in only 27% of patients in the MTH + C group.

### 3.3. Cardiac Interventions

Table 2 lists the cardiac interventions for the MTH + C patient population. Early cardiac catheterization (<12 hrs) was performed in the majority (86%) of patients. Although the overall mean time to catheterization from ROSC was 290 ± 333 minutes, STEMI patients received emergency coronary angiography sooner than patients without ECG changes (212 ± 94 minutes versus 465 ± 140 minutes). PCI was performed in two-thirds of patients and successful PCI was achieved in the majority of these cases (89%).

### 3.4. Outcomes

In patients who underwent MTH with and without cardiac catheterization, the in-hospital mortality was 48% and 78%, respectively. Among survivors in the MTH + C group, only 48% of patients (32/66 patients) had good (CPC 1-2) neurologic recovery. On the contrary, among survivors in the MTH + NC group, only 13% of patients (14/110 patients) had good (CPC 1-2) neurologic recovery. Of those who survived in the MTH + C group, 20% patients were transferred to rehabilitation or referring hospital and one-third of the patients were successfully discharged home (Table 3). In the MTH + NC group, only 5% were transferred to a long-term facility, and only 3% were successfully discharged home.

### 3.5. Predictors of In-Hospital Mortality

Univariate predictors of in-hospital mortality for patients with MTH + C included increasing age, diabetes, dyslipidemia, baseline renal insufficiency postcardiac arrest, cardiogenic shock, use of dobutamine, and failure to initiate MTH prior to catheterization (Table 4). Multivariate analysis of these predictors identified baseline renal insufficiency as the only independent predictor of in-hospital mortality in patients with MTH + C (OR = 8.2, 95% CI 1.8–47.1, *p* = 0.009). In patients with MTH + NC, shock was the only independent predictor of in-hospital mortality (OR = 3.5, 95% CI 1.0–11.3, *p* = 0.04).

### 3.6. Predictors of Neurologic Outcome

Diabetes, absence of immediate bystander CPR, increased collapse to ROSC time, baseline renal insufficiency, lower baseline pH, postcardiac arrest seizure, and failure to initiate MTH prior to catheterization were univariate negative predictors of good (CPC 1-2) neurologic outcome in patients with MTH + C (Table 5). Multivariate analysis identified baseline renal insufficiency (OR = 0.15, 95% CI 0.02–0.71, *p* = 0.03) and the absence of immediate bystander CPR (OR = 0.22, 95% CI 0.05–0.9, *p* = 0.04) as independent negative predictors of good neurologic outcome in patients with MTH + C. Univariate analysis for predictors of neurological outcome in patients with MTH + NC was not performed due to the small sample size of* n* = 10 who had good neurological outcome (CPC 1-2) in this study group.

## 4. Discussion

The current study describes the outcomes and predictors of increased mortality for patients resuscitated from cardiac arrest and treated with the combination of MTH and catheterization versus MTH with no cardiac catheterization. In-hospital mortality for the study population of MTH + C was high at 48%, with 97% of patient mortality attributed to brain death, as is typical for patients with ROSC [1, 3, 4]. Baseline renal insufficiency following resuscitation from cardiac arrest was determined to be the only independent predictor of in-hospital mortality in patients with MTH + C. Furthermore, independent negative predictors of neurologic outcome were determined to be baseline renal insufficiency and absence of immediate bystander CPR for patients with MTH + C.

Neurological outcomes after cardiac arrest are traditionally dismal. The poor prognosis is attributed to postcardiac arrest syndrome, which encompasses systemic ischemic-reperfusion injury with subsequent biochemical, structural, and functional insult [17, 18]. Ultimately, this leads to progressive cell destruction, postcardiac arrest brain injury, circulatory dysfunction, multiorgan failure, and death [17, 18]. The beneficial effects of MTH are based on the prevention of this cascade, specifically, by reducing cellular metabolic needs and inhibiting temperature-sensitive pathways of the ischemia-reperfusion cascade to slow ongoing hypoxic neurological insult [13, 17, 18]. As such, a number of studies have demonstrated improved neurological outcomes with the use of MTH in the postcardiac arrest period. In a multicentre blinded randomized control trial involving patients resuscitated after cardiac arrest due to VT, 55% patients randomized to receive MTH had a favorable neurologic outcome, compared to 39% in the control group [5]. A recent study by Neilsen et al. also demonstrated that targeted temperature management at 36°C after OHCA conferred a similar benefit to MTH from a neurological function standpoint [6]. Our study corroborates that individuals who survive aggressive postresuscitative care are neurologically intact, with 48% of survivors having favorable neurological function at hospital discharge. The preservation of neurological function is an important treatment goal, as survival is correlated with neurologic status [13].

In addition to MTH, the role of early invasive strategies for postcardiac arrest patients has gained recent attention. In previous studies, 40–57% of OHCA patients without ST-segment elevation had pathological findings with therapeutic options on coronary angiography [11, 19]. A recent retrospective study involving a cohort of 435 patients with OHCA of presumed cardiac origin reported the poor predictive value of ST-segment elevation for coronary occlusion in the setting of cardiac arrest [11]. Furthermore, successful immediate coronary angioplasty was associated with a survival benefit regardless of ECG findings, suggesting that immediate catheterization may be warranted in the setting of resuscitated cardiac arrests even in the absence of ST-segment elevation [11]. This has led to an increased adoption of emergency coronary angiography for all patients with OHCA of suspected cardiac origin [20, 21]. In the current study of patients who underwent MTH + C, 32% did not have ST-segment elevation, 32% did not undergo PCI, and 18% had angiographically normal coronary arteries or nonocclusive coronary artery disease. Only patients with ST-segment elevation underwent emergent PCI after invasive angiography, raising question to the use of universal emergent angiography postcardiac arrest. This is an important consideration as there are inherent risks associated with coronary angiography and the ultimate goal is to reduce the number of patients undergoing an unnecessary procedure.

A contemporary approach to coordinated postresuscitative care by combining MTH with coronary angiography is associated with more favorable patient outcomes [13, 22, 23]. Sunde et al. reported 56% survival with favorable neurologic outcomes for patients randomized to a standardized postresuscitation protocol involving MTH and PCI, as compared to 26% in the control treatment arm [22]. Similarly, Stub et al. demonstrated that the combination of therapeutic hypothermia with early coronary intervention was associated with an improved survival of 64%, as compared to 39% in the control group [13]. The results of the current study are comparable to those previously stated. However, in our study over 80% of patients experienced cardiogenic shock within the first week after cardiac arrest and 36% required intra-aortic balloon pump counterpulsation, which is significantly higher than that reported in the previous literature [13]. Unlike previous studies, we did not exclude cardiogenic shock patients from the MTH protocol [5, 6, 9]. Although circulatory shock is still considered a relative contraindication for MTH, several recent studies including ours suggest that these patients may still derive benefit from MTH [24, 25].

Previous literature has identified key clinical characteristics to predict survival from OHCA: initial location of OHCA, witnessed cardiac arrest, prompt bystander CPR, ECG with shockable cardiac rhythm, early defibrillation, time to resuscitation, complete revascularization, hyperlactatemia, and presence of ABI [4, 6, 26–30]. In addition, renal insufficiency is a known predictor of mortality in the cardiogenic shock population [30]. Kidney function as assessed by CrCl appears to be a sensitive marker of poor tissue perfusion during cardiac arrest and perhaps an indirect marker of poor cerebral perfusion. Our findings confirmed the importance of renal function as an independent predictor of both in-hospital mortality and neurological outcome in the OHCA patient population who underwent MTH + C.

In addition to renal perfusion, bystander CPR improved 1-year survival with favorable neurological outcomes for OHCA patients in a study by Iwami et al. [29]. A similar study by Herlitz et al. further supported the survival benefit of bystander CPR afforded to this patient population [28]. Prompt provision of CPR may delay the degradation of tachyarrhythmias to asystole, explaining the positive impact on survival. Despite the majority of patients having experienced witnessed cardiac arrest, only half of patients had immediate bystander CPR in our population. The absence of immediate CPR was a potent independent negative predictor of good neurologic recovery, in keeping with previous studies. This stresses the critical importance of public awareness and education in early bystander CPR to improve the neurologic outcome for OHCA patients. The study population also experienced a longer median arrest time (low flow) (28 ± 15 min) as compared with other studies [5, 6]. A longer interval from collapse to ROSC has been associated with unfavorable neurologic outcomes, which is corroborated by our findings [6].

Although the correct population was cooled in the current study, the time to reach target temperature (32–34°C) from initiation of MTH was relatively prolonged at 8 hrs. The prolonged time to reach target temperature may be attributable to delayed initiation of the MTH protocol as a consequence of early coronary angiography, as less than 25% of our study population was cooled prior to catheterization. Of note, there was a trend towards less favorable outcomes in patients who had no cooling prior to coronary angiography, and it was a univariate predictor of in-hospital mortality and neurological outcome. This suggests the need for a well-planned and coordinated cooling protocol which is implemented prior to and during catheterization, such that early catheterization does not delay the initiation of MTH. Second, a transport based MTH protocol is required, in order to enable the implementation of MTH en route to central catheterization facilities. Finally, increased education for earlier MTH implementation in peripheral facilities (ER, ICU) will hopefully improve patient outcomes.

There are several limitations to the current study. This is a single-centre retrospective review and thus subject to potential confounders and selection bias. Data collection was limited by available documentation and not all measurements were made at exactly the same time intervals or course in hospital. We used the CPC score to assess neurologic recovery because of its ease of use and widespread reporting in the literature. However, this scoring system is not well validated and was retrospectively assigned based on clinical documentation at the time of discharge. Finally, our study does not include long-term outcome data and was limited to hospital discharge.

## 5. Conclusion

Although the outcome for patients resuscitated from cardiac arrest is traditionally low, it may be improved by the use of coordinated resuscitation protocols involving MTH and cardiac catheterization. Baseline renal insufficiency appears to be a potent predictor of in-hospital mortality and poor neurologic outcome in this population. The absence of immediate CPR also predicts poor neurologic recovery and this should be an impetus for widespread public education. Further multicentre registry collaboration may be indicated to study and refine outcomes in this severely ill patient population.

## Figures and Tables

**Table 1:** Baseline clinical characteristics, prehospital arrest data, and cooling protocol of total population (*n* = 176).

Clinical characteristics	MTH + C (*n* = 66)	MTH + NC (*n* = 110)	*p* value
Age (yrs)	61 ± 12	61 ± 16	1.00
Sex (M)	52 (79)	77 (70)	0.22
Medical history			
Diabetes (%)	12 (18)	34 (31)	0.06
Smoking (%)	36 (55)	49 (45)	0.22
Hypertension (%)	36 (55)	65 (59)	0.63
Dyslipidemia (%)	27 (41)	39 (35)	0.42
Prior MI (%)	22 (33)	32 (29)	0.32
Prior PCI (%)	5 (8)	7 (6)	0.63
Baseline CRI (%)	17 (26)	12 (11)	0.24
Cardiac arrest			
OHCA (%)	50 (76)	89 (81)	0.44
Witnessed OHCA (%)	61 (92)	83 (75)	0.07
Immediate bystander CPR (%)	36 (55)	40 (45)	0.20
Duration of bystander CPR (min)	6.4 ± 4.1	7.5 ± 4.0	0.08
Time to ROSC from collapse (min)	28.0 ± 14.6	24.0 ± 14.8	0.10
Time from collapse to EMS (min)	8.0 ± 5.0	12.0 ± 12.0	<0.05
Total cooled time (min)	1671 ± 410	1625 ± 551	0.62
Initial arrest rhythm			
VF/pulseless VT	61 (92)	39 (35)	<0.05
PEA	5 (8)	61 (65)	<0.05
STEMI	45 (68)	0 (0)	<0.05
Mild therapeutic hypothermia			
Time from ROSC to cooling (min)	277 ± 110	211 ± 146	<0.05
Time to achieve 32–34°C from cooling (min)	252 ± 174	312 ± 466	0.30
Total cooled time (min)	1671 ± 410	1625 ± 551	0.56

Values are mean ± SD or *n* (%). MTH + C, mild therapeutic hypothermia with cardiac catheterization; OHCA, out of hospital cardiac arrest; MTH + NC, mild therapeutic hypothermia with no cardiac catheterization; yrs, years; m, males; MI, myocardial infarction; PCI, percutaneous coronary intervention; CRI, chronic renal insufficiency; CPR, cardiopulmonary resuscitation; min, minutes; ROSC, return of spontaneous circulation; EMS, emergency medical services; VF, ventricular fibrillation; VT, ventricular tachycardia; PEA, pulseless electrical activity; STEMI, ST elevation myocardial infarction.

**Table 2:** Cardiac catheterization findings in study population who underwent MTH + C (*n* = 66).

Cardiogenic shock	53 (80)
Vasopressors	53 (80)
Inotropes	26 (40)
Duration of support (min)	52 ± 73

IABP use	24 (36)

ECMO use	3 (5)

Early catheterization (<12 hrs)	56 (86)

Time to catheterization from ROSC (min)	290 ± 333
STEMI (min)	212 ± 94
No STEMI (min)	465 ± 140

(1) Vessel CAD	24 (36)
(2) Vessel CAD	16 (24)
(3) Vessel CAD	14 (21)
Branch vessel disease or no culprit	12 (18)

PCI	45 (68)

Multivessel PCI	10 (22)

Stent deployment	43 (96)

Number of stents utilized	1.7 ± 1.1

Stent thrombosis	2 (4)

Successful PCI	40 (89)

Mean TIMI flow pre (min)	1.4 ± 1.4
Mean TIMI flow post (min)	2.9 ± 0.6

CABG	2 (3)

GPIIbIIIa inhibition	23 (51)

Values are mean ± SD or *n* (%). IABP, intra-aortic balloon pump; ECMO, extracorporeal membrane oxygenation; ROSC, return of spontaneous circulation; STEMI, ST elevation myocardial infarction; CAD, coronary artery disease; PCI, percutaneous coronary intervention; TIMI, thrombolysis in myocardial infarction; CABG, coronary artery bypass grafting.

**Table 3:** In-hospital outcomes for total population (*n* = 176).

Clinical characteristics	MTH + C (*n* = 66)	MTH + NC (*n* = 110)	*p* value
In-hospital mortality (%)	32 (48)	86 (78)	<0.05
Discharged home (%)	21 (32)	3 (3)	<0.05
Discharged to long term facility (%)	12 (20)	5 (5)	<0.05
CPC 1-2 neurological recovery (%)	32 (48)	10 (9)	<0.05
CPC 3–5 neurological recovery (%)	34 (52)	100 (91)	<0.05
Length of hospital stay (days)	12 ± 14	8 ± 8	<0.05
Length of ICU stay (days)	7 ± 6	5 ± 5	0.35

Values are mean ± SD or *n* (%). MTH + C, mild therapeutic hypothermia with cardiac catheterization; MTH + NC, mild therapeutic hypothermia with no cardiac catheterization; CPC, cerebral performance category; ICU, intensive care unit.

**Table 4:** Univariate predictors of in-hospital mortality for study population (*n* = 176).

Clinical characteristics	MTH + C (*n* = 66)	MTH + NC (*n* = 110)
Age	0.04	0.4
Diabetes	0.02	0.4
Dyslipidemia	0.02	0.6
Baseline CRI	0.003	0.6
Cardiogenic shock	0.05	0.04
Absence of cooling prior to cardiac catheterization	0.04	N/A
Use of dobutamine	0.04	0.06

MTH + C, mild therapeutic hypothermia with cardiac catheterization; MTH + NC, mild therapeutic hypothermia with no cardiac catheterization; CRI, chronic renal insufficiency.

**Table 5:** Univariate negative predictors of good neurologic outcome in study population who underwent MTH + C (*n* = 66).

Variables	*p* value
Diabetes	0.03
Absence of immediate CPR	0.03
Collapse to ROSC time	0.02
Baseline renal insufficiency	0.006
Baseline pH	0.03
No cooling implemented before catheterization	0.02
Seizure	0.007

MTH + C, mild therapeutic hypothermia with cardiac catheterization; CPR, cardiac pulmonary resuscitation; ROSC, return of spontaneous circulation.
